# Comprehensive Health Challenges and Management Strategies for International Business Travelers: A Scoping Review

**DOI:** 10.31662/jmaj.2025-0085

**Published:** 2025-09-05

**Authors:** Yayoi Tetsuou Tsukada, Ritsuko Okamura, Masahiro Yasutake

**Affiliations:** 1 Department of General Medicine and Health Science, Nippon Medical School, Tokyo, Japan; 2 Department of General Medicine, Nippon Medical School Musashi Kosugi Hospital, Kanagawa, Japan

**Keywords:** overseas business travel, Japan, occupational health, international business travel

## Abstract

**Background:**

The globalization of economic activities has led to a significant increase in international business travelers (IBTs), exposing them to various health risks, including infectious diseases, physical and psychological stress, and inadequate access to medical care. However, comprehensive research on IBTs’ health remains limited. This study aims to assess IBTs’ health concerns through a scoping review, focusing on illnesses during travel, the impact on daily life and well-being, and corporate health management.

**Methods:**

A scoping review was conducted following the Arksey and O’Malley framework and the Joanna Briggs Institute methodology. Extensive searches were performed in MEDLINE, Web of Science, and Ichushi-Web, targeting studies for studies published between 2013 and 2022. Eligible studies addressed IBTs’ physical and mental health or healthcare access. A two-step screening process was applied, and relevant data were extracted and categorized.

**Results:**

A total of 31 studies were included. Health risks for IBTs were classified into three categories: (1) travel-related illnesses, including infectious diseases and medical emergencies; (2) physical and psychological health impacts, such as sleep disturbances, obesity, and mental stress; and (3) corporate health management strategies. Limited pre-travel consultations and vaccinations were notable concerns, particularly among Japanese IBTs.

**Conclusions:**

IBTs face significant health risks that require improved prevention strategies, including vaccination programs, corporate health policies, and psychological support. As global business travel resumes post-pandemic, comprehensive health management tailored to IBTs’ needs is essential to ensure their well-being and operational efficiency.

## Introduction

The globalization of economic and social activities has led to a significant increase in international business travelers (IBTs). The number of IBTs worldwide surged from 642 million in 1980 to approximately 3.4 billion in 2019. In Japan, this figure grew from 127,000 in 1964 to approximately 20 million in 2019 ^(1), (2)^. Business and professional travel accounts for 14% of all international tourist arrivals globally ^(3)^ and 12.3% in Japan ^(2)^.

IBTs face numerous challenges, including long-distance travel, jet lag ^(4), (5), (6), (7)^, psychological burdens associated with work responsibilities ^(8), (9)^, environmental and cultural differences, and limited access to medical care. However, the health concerns of IBTs remain less understood than those of expatriates ^(10),(11)^. Additionally, the strain between work and family life caused by assignments without accompanying family members has drawn increasing attention ^(12),(13)^.

Existing studies on IBTs’ health primarily focus on specific risks encountered during business trips, such as infectious diseases (e.g., malaria, traveler’s diarrhea, sexually transmitted diseases) and travel-related trauma. Despite the substantial physical and psychological stress IBTs endure, comprehensive medical care for this group has been insufficiently documented. This gap highlights the need for more detailed research into the health impacts of frequent business travel ^(11)^.

One challenge in studying short-term IBTs is the difficulty of tracking health trends over brief periods. In Japan, expatriate employees assigned abroad for six months or longer are monitored through visa applications, and overseas resident officers are required to undergo medical examinations before departure and upon return under Japanese law. However, short-term IBTs are not legally obligated to notify the Ministry of Foreign Affairs or undergo health screenings under the Industrial Safety and Health Act (Act Number 57 of 1972), making it impossible to determine their exact numbers or health status. Some studies have used health insurance claims from multinational corporations to investigate IBT health issues, but these are limited to single organizations, restricting the generalizability of findings ^(14), (15), (16)^.

The coronavirus disease 2019 (COVID-19) pandemic severely impacted international business travel, reducing the number of IBTs to 3.1 million in 2020 and 0.5 million in 2021 ^(1)^. The pandemic disrupted IBTs’ activities through travel restrictions, mandatory Polymerase Chain Reaction testing, quarantine measures, varying international infection control policies, risk of infection abroad, and disparities in healthcare quality across destinations.

In September 2022, business travel resumed following the decline in infections. Ensuring IBTs’ health is critical to minimizing unexpected medical issues that could disrupt corporate operations and finances. Japan, as an island nation, relies solely on air travel, which poses additional health risks due to long flights, extensive boarding times, and significant time zone differences. Despite these challenges, travel to neighboring Asian countries, where infectious disease risks remain high, has increased. Prior to the pandemic, IBTs and their employers demonstrated limited awareness of health precautions during overseas travel ^(17), (18), (19)^. Among Japanese IBTs, rates of pre-travel medical consultations and vaccinations were notably low ^(17)^.

The persistence of pandemics may reduce travel frequency in some sectors, as online conferencing ^(2), (20), (21)^ increasingly replaces in-person meetings. Moreover, the challenges IBTs face may vary depending on company size. As a result, global corporations must reassess and strengthen health management strategies for IBTs in the post-pandemic era ^(22), (23), (24)^. Given IBTs’ critical role in the global economy, their health concerns must be addressed as a global issue, rather than solely by individual companies. Understanding business travel trends and emerging health risks is essential.

In 2021, we conducted a questionnaire survey to assess prevailing trends and emerging challenges associated with overseas business travel among Japanese listed companies, with a particular focus on the health concerns of IBTs. To support the development and evaluation of this questionnaire survey, we conducted a scoping review of existing studies on health issues among IBTs and examined broader research trends pertaining to this population.

## Materials and Methods

### Scoping review

This scoping review was conducted following the methodological framework introduced by Arksey and O’Malley (2005) and aligned with the Joanna Briggs Institute methodology ^(25)^. We adhered to the Preferred Reporting Items for Systematic Reviews and Meta-Analyses extension for scoping reviews checklist ^(26)^. We aimed to answer the following research questions: 1. What are the health-related issues for short-term IBTs (6 months or less)? 2. What are the current and future research topics in this area? Using these research questions as guidance, we engaged in an iterative process that involved searching the literature, identifying search terms, and developing and refining our search strategy to identify relevant publications.

### Search strategy

The databases searched were MEDLINE, Web of Science, and Ichushi-Web (Japanese Database). The search terms were developed through a pilot search and prior knowledge of the subject area, and a consensus-building process to identify keywords from existing literature. Search formulas were created using the synonyms “overseas business trip,” “industry,” “occupation,” “hygiene,” “health,” and “medicine,” included in the title or on the form, with the intention that medical and health issues would be extracted. The search formula was as follows: (((industrial) OR (occupational)) AND ((medicine) OR (health) OR (hygiene))) AND ((((overseas business travel) OR (business travel abroad)) OR (international business travel)) OR (((overseas business trip) OR (business trip abroad)) OR (international business trip)))

In addition to a free-text word search, MEDLINE subject headings were also targeted. The search was conducted in January 2023 and was limited to publications from the 10 years prior to the survey to 2022.

### Eligibility criteria

A report was eligible for review if it met all the following inclusion criteria:

1. It is an original research study published in a peer-reviewed journal.

2. It addresses the physical and mental health of short-term IBTs.

3. It addresses the healthcare of short-term IBTs.

A report is excluded if it meets one or more of the following criteria:

1. It describes background knowledge of a specific infectious disease or the pathogen itself.

2. It is an epidemiological study, not specific to professional travelers.

3. It is not directly related to the health of the travelers (e.g., studies on carbon dioxide emissions).

4. It describes animal experiments.

5. It pertains to medical tourism.

6. It discusses the development of drugs.

7. It involves the description of mathematical models.

The retrieved literature was evaluated through a two-step screening process. In the primary screening, titles and abstracts were assessed according to the selection criteria. In the secondary screening, the full texts were evaluated based on the same criteria. A team of two researchers (R.O., Y.T.) conducted the literature screening, collaborated closely throughout the process, and held frequent and extensive consensus-building meetings.

### Data management

To ensure maximum objectivity and transparency in the review process, decisions regarding the inclusion or exclusion of studies, along with their rationales, were documented in a digitized spreadsheet. This spreadsheet was used to chart the data systematically. For literature management, EndNote 21 (Clarivate Analytics) was utilized.

### Data extraction and analysis

All included studies underwent a double-blind review by two independent peer reviewers. Each reviewer independently charted data for the following variables: authors, country, year, DOI, study design, study objective, population studied, outcome, main findings, and data analysis. To facilitate data integration, all studies under review were meticulously charted by the reviewers.

## Results

### Scoping review

**Figure 1.**
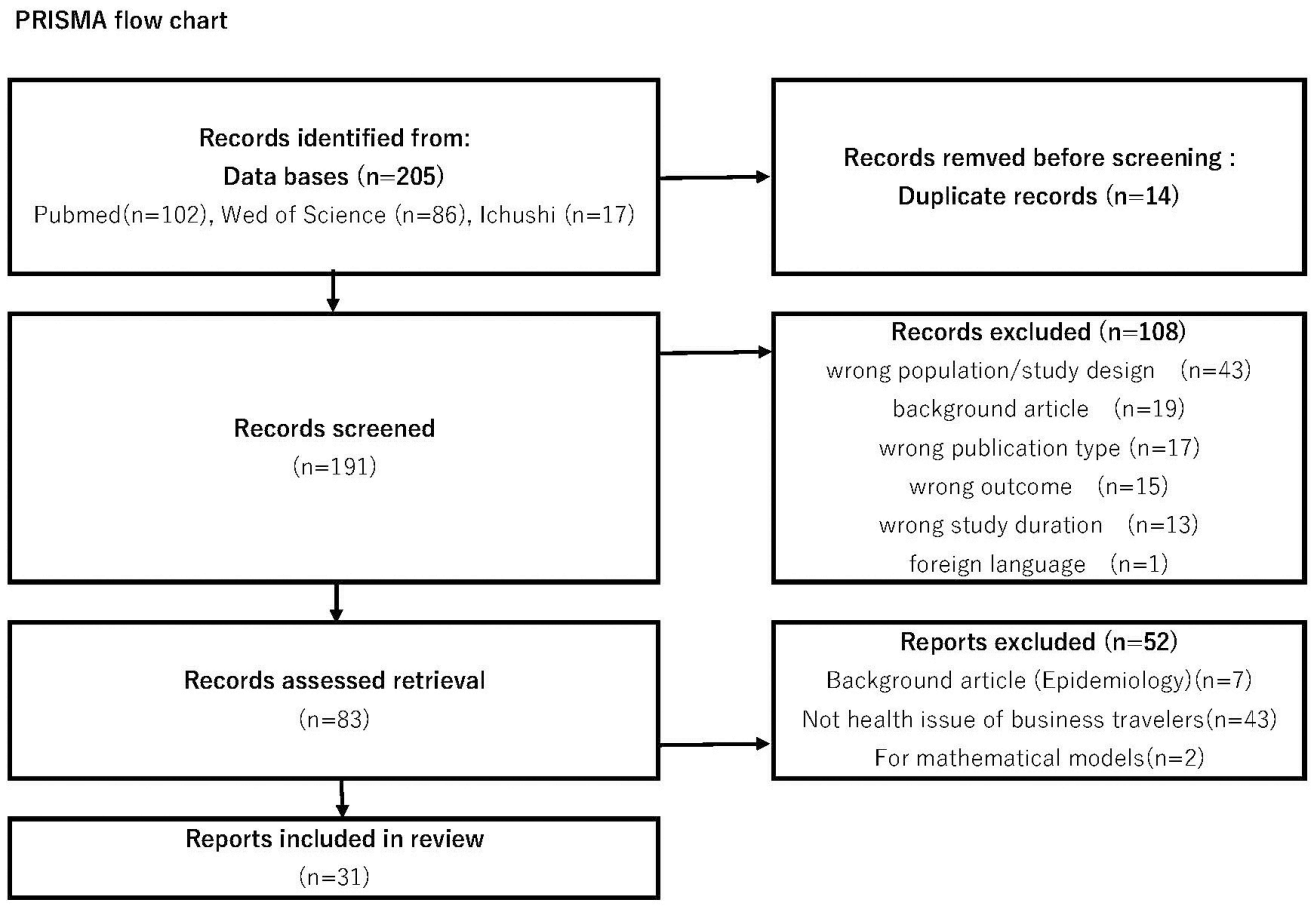
PRISMA flow chart. PRISMA: Preferred Reporting Items for Systematic Reviews and Meta-Analyses.

The search resulted in 205 references (102 in PubMed, 86 in Web of Science, and 17 in Icushi-web). After duplicate deletion, 191 records remained for screening. Following the initial screening process, 83 studies were selected for a more detailed examination of their full texts. Ultimately, 31 studies were deemed suitable for inclusion in the final analysis (Figure 1). All results are presented in the summary of findings (Table S1).

These studies were classified into three categories based on their content: “diseases and symptoms incurred during travel and their countermeasures,” “impact of business travel on daily life, physical and mental health,” and “employers'’ health management of IBTs” (Table 1).

**Table 1.** Summary of Scoping Review.

Classification	n	(%)
**Travel-related illnesses and injury n = 17 (53%)**		
Infectious disease (COVID-19)	1	3%
Infectious disease (pre-travel consultation)	3	9%
Infectious disease (Vaccine)	3	9%
Infectious disease (KAP)	3	9%
Jet lag	3	9%
Travel-related illnesses and injury (except for infectious disease)	4	13%
**Impact of business travel on employees’ daily life and well-being n = 12 (38%)**		
Adiposity	1	3%
Well-being	4	13%
Well-being/leadership	1	3%
Work style	2	9%
Work style/ Well-being	3	9%
**Employer healthcare for business travelers n = 3 (9%)**		
**Total**	31	100%

KAP: knowledge, attitudes, and practices of infectious diseases, COVID-19: Coronavirus disease 2019

### Diseases and symptoms incurred during travel and their countermeasures

According to a report of travel-related illnesses among short-term IBTs, the most common diagnoses were acute non-specific diarrhea, viral syndrome, acute bacterial diarrhea, chronic diarrhea, and Plasmodium falciparum malaria (each accounting for less than 10% of cases). Dengue fever was most prevalent in the Caribbean (9%), while dog bites were most common in Eastern Europe (9%). Upper respiratory tract infections were most frequent in Australia, New Zealand, North America, and Western Europe (8%). Acute non-specific diarrhea was the most common diagnosis in the rest of the world (10%-19%). A total of 847 potentially vaccine-preventable diagnoses (7% of all diagnoses) were identified among 1,000 cases. The most frequently reported diagnoses were influenza and influenza-like illnesses (320 cases) ^(27)^.

Another report analyzing 31,860 employees (expatriates and frequent IBTs) of oil companies who had emergency medical evacuation (Medevac) cases abroad found that women were the most frequent users, with digestive disorders, trauma, musculoskeletal disorders, cardiovascular disorders, and neurological disorders. Illness was a more prevalent cause of Medevac than traumatic injury ^(28)^.

It is also clear that business trips to “low” medical risk countries resulted in more hospitalizations and medical transports than trips to “high” medical risk countries. However, business trips to countries with high medical risk carry a much higher risk per trip ^(29)^.

Ten studies on infectious diseases accounted for 59% of the travel-related disease literature and 30% of the total, indicating the importance of infection control for IBTs ^(17), (30), (31), (32), (33), (34), (35), (36), (37), (38)^. The content was divided into three categories: knowledge, attitudes, and practices of infectious diseases in the destination country, vaccine coverage, and use of pre-departure consultation, all related to infection control for the traveler. Vaccine coverage and malaria medication carrying rates differed by source and destination of business travel. Although IBTs had higher vaccination rates than those traveling for other purposes, coverage was still insufficient ^(33), (36), (37)^. In contrast, last-minute travelers (LMTs), who visit a medical institution within a week of departure, are more likely than travelers visiting relatives or friends to be vaccinated, and it is necessary to encourage LMTs to visit a medical institution as early as possible ^(35)^. One report addressed the impact of the COVID-19 pandemic on seafarers. The study revealed that the risk of infection, workplace tensions, and discrimination have had a significant impact on the health and well-being of seafarers. These factors have also contributed to limitations on crew rotation and shore leave, which have a detrimental effect on their physical and mental health ^(30)^.

### Impact of business travel on daily life and physical and mental well-being

Three manuscripts discussed the effects of time differences on performance among IBTs, as well as sleep disturbances, smoking, and physical activity. They highlighted the usefulness of educational programs on managing time differences ^(4), (39), (40)^.

Work style and well-being were explored in four studies on mental health and one on mental health and leadership. Six studies also discussed work style, indicating the health challenges frequent IBTs face. A review of the literature revealed a paucity of studies on the general health challenges of frequent travel, highlighting the difficulty of managing the health of IBTs. One report found associations between the frequency of business travel and health behaviors and adiposity in businessmen ^(41)^.

Recent studies have increasingly concentrated on the mental health implications for IBTs. A 2018 U.S. report found elevated rates of smoking, insomnia, physical inactivity, alcohol abuse, anxiety, and depression among frequent IBTs ^(8)^. Similarly, a 2021 Finnish report indicated that more days spent on international business travel correlated with higher job fatigue. Moreover, leadership quality was linked to both work and travel fatigue ^(42)^. A 2020 UK structured interview study suggested that travel load and intensity influence work-related outcomes such as stress and well-being, with these effects moderated by cognitive and behavioral characteristics ^(43)^. These findings suggest a framework for improving IBTs’ well-being by managing job and travel characteristics and providing training to enhance self-management skills. The frequency and intensity of business travel impact work outcomes, including stress and health status. Further, an analysis of social networking responses to studies on IBTs’ health showed mixed reactions, with some denying the health impact of frequent travel and others calling for public health warnings ^(44)^.

In a different context, a 2017 Japanese report highlighted that pilots experience varying levels of fatigue depending on flight distance, with flight duration, work schedules, and sleep deprivation identified as significant predictors of fatigue ^(45)^.

### Work-family conflict among IBTs

Two UK surveys found no direct association between work-family conflict (WFC) and health problems, but emotional fatigue mediated the relationship between WFC and health outcomes. Similar patterns were observed among weekly commuters, domestic travelers, and IBTs. The association between emotional fatigue and musculoskeletal pain was significantly stronger in commuters who return home only on weekends and spend weekdays at their assignment sites than in domestic and international travelers ^(12)^.

In another survey for WFC, 13% of both sexes reported that WFC limited their business travel. Additionally, 29% of men and 17% of women reported that their work performance was affected during business travel, with frequent IBTs being more likely to report performance impacts due to WFC ^(46)^.

### Employer health management for IBTs

Recent studies have reported on the use of online conferencing and business travel ^(47), (48), (49)^. All three reports on corporate health management came from Japanese occupational physician groups. Japan’s geographical distance from Europe and the U.S., and its corporate culture of generosity to employees, may distinguish its approach from that of other countries. However, short-term business trips do not require legal control by companies, which has limited the implementation of internal health policies for IBTs

## Discussion

This study conducted a scoping review to gain insight into the health issues of short-term IBTs and the impact of COVID-19 on short-term IBTs. The scoping review identified three main health issues related to short-term IBT in recent years. These categories are as follows: 1) illnesses and symptoms that develop during travel and the measures to address them, 2) the impact of business travel on daily life and mental and physical health, and 3) employer health management for IBTs.

### Trends in overseas and Japanese travel

In Japan, as in other countries around the world, the quarantine restrictions related to COVID-19 were gradually lifted, and the country began to accept foreign tourists again. On September 4, 2022, the Japanese government lifted all landing restrictions on individuals traveling to Japan who were previously subjected to landing denial. Visa exemption arrangements resumed from 0:00 a.m. (JST) On October 11, 2022. From 0:00 a.m. (JST) On April 29, 2023, all IBTs and returnees were no longer required to submit either a certificate of a negative COVID-19 test result conducted within 72 hours prior to departure or a valid COVID-19 vaccination certificate of three doses or equivalent ^(50)^. Thus, international tourism demonstrated a strong recovery through July 2022, with arrivals reaching 57% of pre-pandemic levels in the first seven months of 2022. International tourist arrivals almost tripled (+172%) in January-July 2022 compared to the same period in 2021 ^(1)^. Moreover, ICT-based alternatives such as online conferencing have become widely available. It is now easier to hold meetings and conferences without having to travel directly to a site. However, while business travel has declined, it remains essential in the post-COVID-19 era.

### Communicable diseases in short-term IBTs

Measures to prevent infectious diseases have become a major challenge for companies during the COVID-19 pandemic. In the context of international travel, vaccinations and pre-departure consultations are essential. However, their importance has often been neglected, despite various initiatives reported for preventable infectious diseases among IBTs ^(33), (36), (37), (38)^. In Japan and other countries, a common issue with IBTs is the tendency for people to rush health check-ups at the last minute ^(33), (51)^. Executives of global companies may need to travel abroad suddenly due to business emergencies. Conversely, many vaccinations require several doses given at intervals of one to four weeks, which takes time. Recent efforts have aimed to shorten vaccination intervals for diseases. Under normal circumstances, the Japanese hepatitis A vaccine (Aimgen) requires a primary series of three doses. However, certain other inactivated vaccines, such as Havrix, have been approved by the U.S. FDA for use as a single-dose regimen, with protective antibody levels typically achieved approximately 14 days after administration; subsequent booster doses are recommended at 6-12 months ^(52)^. For Japanese encephalitis, although three doses are generally required, a combination vaccine that includes hepatitis B is also available. Additionally, IXIARO, a Japanese encephalitis vaccine approved by the U.S. FDA, offers a two-dose regimen for adults, with the second dose administered one week after the first. Protective antibody levels can be achieved within one week of completing this course ^(53)^. Similarly, rabies vaccination traditionally involves three doses, but the World Health Organization now recommends an alternative two-dose regimen administered at one-week intervals ^(54)^. By leveraging these imported vaccines and alternative immunization schedules, it is possible to reduce the total vaccination period from over one month to approximately two weeks, if immunization is initiated at least two weeks prior to departure. To safeguard both health and business opportunities, it is crucial for businesspeople in relevant departments of global companies to ensure they receive necessary vaccinations individually. A comprehensive personal electronic health record, which includes vaccination records, would be highly beneficial ^(55), (56), (57)^.

### The impact of international business travel on workers’ physical health

The scoping review identified only one relevant report of the impact of IB Travels on Workers’ Physical Health ^(41)^. This report indicated that, after accounting for age, exercise, and sleep, the total number of domestic and international trips was more strongly associated with body mass index, body fat percentage, and visceral adipose tissue in women than in men. Among men, the frequency of international travel had a greater impact on obesity compared to the total number of domestic trips taken. A 2011 report from before the period of review found that individuals who did not travel for business were more likely to report poor health and discomfort compared to those who traveled infrequently (1-6 nights per month) ^(58)^. As the frequency of business trips increased, the rate of health complaints also increased, reaching 2.61 for those who traveled frequently (20 or more nights per month). The odds ratio for obesity was highest among those who never traveled compared to those who traveled frequently. Conversely, an analysis of health risk assessment records of 12,942 American employees of multinational companies in 2010 showed that international business travel was significantly associated with a lower body mass index and lower blood pressure ^(9)^. In general, social jet lag, often observed in shift workers, is associated with metabolic syndrome and cardiovascular events ^(59), (60), (61), (62)^. To date, no analysis has been conducted on the frequency of business trips, obesity, or metabolism in the Japanese population. Further, most of the existing reports are analyses of a single company. There is insufficient evidence to establish a relationship between short-term business trips and lifestyle diseases. A detailed study is needed using claim data from various companies, categorized by industry type, company size, and nationality.

This review identified several factors that contribute to jet lag-related fatigue, including travel distance, direction of travel (particularly eastward journeys), the availability of opportunities for napping during work hours, and the adequacy of rest intervals between shifts. It was also noted that many IBTs adopt non-pharmacological strategies to mitigate jet lag, often relying on information obtained from colleagues, family members, or the internet.

To alleviate the physical burden associated with time zone differences among IBTs, we recommend that companies: 1) Optimize the frequency of overseas assignments, ensure sufficient intervals between trips, and implement systems that allow for rest or naps; and 2) Provide educational programs incorporating evidence-based strategies for circadian adjustment.

### The impact of international business travel on workers’ mental health

In recent years, ample attention has been paid to the psychological stress experienced by frequent IBTs. This review included nine studies, such as the sensationally titled “The Dark Side of Business Travel: A Media Comments Analysis” ^(44)^. Business travelers are often executives or leaders at higher levels, who travel to various locations on multiple short-term business trips without their families and tend to stay in temporary accommodation while working across dual or multiple work contexts for short periods of time. Owing to the high demands of their jobs, the pressure on them from their organizations is inevitably high. The aforementioned report found that poor physical health was significantly associated with a loss of confidence in one’s ability to maintain the pace of work ^(45)^. Additionally, unlike expatriates, IBTs who do not bring their families with them face the challenge of balancing work and family life, which can also be a source of stress. A significant correlation between the number of business trips and the difficulty of balancing work and family life has also been reported ^(13)^. Consequently, in recent years, effective use of human resources and employee assistance programs has been proposed as solutions to address this issue. Recent reports have highlighted effective strategies for reducing the emotional stress of telecommuting employees during the pandemic ^(62)^. These strategies include providing timely information, implementing employee assistance programs, fostering informal communication channels, and strengthening COVID-19 management measures as part of broader organizational support ^(9)^. These approaches address both the informational and emotional needs of employees, enhancing their well-being during periods of infection and in post-return quarantine ^(8), (24), (63), (64)^.

### Employer health management for IBTs

Studies on the health management of IBTs were derived from a group of Japanese occupational physicians ^(47), (48), (49)^. In Japan, the Occupational Health and Safety Law mandates that employees dispatched overseas for six months or more must undergo a medical examination before and after their deployment. However, there is no such obligation for short-term IBTs, and the management of pre-departure medical examinations is left to the discretion of each company. Health education, vaccinations, and other measures to prevent infectious diseases before travel are optional. For example, some companies provide pre-travel counseling for employees prior to overseas business trips ^(35), (36), (37)^ and distribute travel kits that include items such as analgesics and gastrointestinal medications ^(65)^.

Workers’ accident compensation insurance and public medical insurance in Japan cover some costs, but the scope is limited, and the compensation amounts are low due to Japan’s low-cost medical system. Not only in developing countries with high health risks but also in countries with low health risks, expensive medical care can impose a considerable financial burden. Protecting the health of IBTs is not only a matter of corporate social responsibility and risk management but also a crucial issue for the employees themselves. Many companies, in addition to providing coverage through Japan’s national health insurance and local medical insurance at the assignment location, also purchase overseas travel or medical insurance to cover costs not reimbursed by Japanese health insurance. This approach helps mitigate the risk of employees having to bear the full cost of medical expenses out of pocket.

Although short-term IBTs are more common than long-term IBTs, their health issues have not been fully investigated, and existing measures are insufficient. It is recommended that companies, based on their industry and organizational context, provide IBTs with timely information on infectious diseases and local medical institutions. Furthermore, employees should be required to enroll in overseas medical insurance, receive consultations with occupational physicians or public health nurses before and after travel, and undergo routine medical examinations along with appropriate post-travel follow-up. In addition, it is desirable to establish a comprehensive personal electronic health record system that includes vaccination history.

Global companies need to consider general occupational health management and industrial hygiene for IBTs. Some international medical networks offer travel clinics for IBTs and their members. Notably, industrial physician training curricula in Japan, including the Japan Medical Association Certified Industrial Physician Training Course and the program offered by the Japan Society of Travel Medicine Industrial Physician Training Course, incorporate mandatory training on health management for overseas assignees. During pandemics, more careful measures to prevent infection, such as vaccination and proof of vaccination, are needed. Companies have been required to implement new measures since the outbreak of COVID-19, including understanding the infection situation in the destination country, formulating return-to-work policies for employees returning from overseas, and implementing cluster measures in the workplace ^(66), (67)^.

During the pandemic, various rumors about prevention and treatment circulated through social media and other channels, causing confusion in the workplace. To prevent this, it is essential for the government and experts to maintain public health responsibly by providing accurate risk communication to companies and citizens during a pandemic. Companies should adhere to these guidelines and manage their workplaces appropriately from the perspective of industrial hygiene, including measures related to business travel ^(68)^. Specifically, four key areas should be addressed: (1) early access to essential information; (2) timely responses to emerging “infodemics”; (3) identification and mitigation of barriers to sustained engagement; and (4) the development of effective communication strategies ^(68)^. Companies will also need to ensure that they have appropriate medical officers in place to manage health-related issues effectively ^(69)^. Providing accurate information to IBTs and ensuring their safety is vital for both the government and companies.

In the event of an outbreak of an unknown infectious disease, particularly a new strain of influenza, new measures will be required. These measures include understanding the infection situation in the destination country, formulating a policy for returning employees from overseas, and implementing cluster measures in the workplace. The Ministry of Health, Labour and Welfare has formulated “Guidelines for Workplaces and Offices Regarding Measures Against New Strains of Influenza.” In preparation for future pandemics, it is recommended that companies develop countermeasures in advance according to the stage of the pandemic ^(70)^. As international business travel resumes, it may be necessary to review and strengthen comprehensive health management strategies for IBTs.

### Limitations

This scoping review focused on the decade preceding the implementation of the survey, with the goal of ensuring consistency with the questionnaire. Reports on the impact of COVID-19 have surged following the analysis period, necessitating a future summary. Additionally, while the review concentrated on IBTs, it proved challenging to entirely segregate international travel by purpose. Notably, reports on epidemiology and specific infections included IBTs as participants, but some did not analyze the purpose of the trip, leading to their exclusion.

### Conclusions

In this study, the key issues identified for international business travelers included, (1) diseases and symptoms incurred during travel and their countermeasures; and (2) the impact of business travel on daily life, as well as physical and mental health. To effectively address these challenges, proactive and comprehensive health management by employers is essential, specifically through structured and sustained approaches to employer health management of IBTs.

As international business travel resumes, we would also like to emphasize the critical role of employers, occupational physicians, and occupational health nurses. Employers are expected to establish organizational systems that promote pre-travel risk assessments, timely access to medical care during travel, and post-travel follow-up. Occupational physicians and health nurses are encouraged to take an active role in educating employees, monitoring health conditions before and after business trips, and contributing to the development of evidence-based workplace policies that support the well-being of IBTs, who play a vital role in the global community.

## Article Information

### Acknowledgments

The authors thank Ms. Naoko Maekawa for providing administrative support. We also acknowledge the use of generative AI for English proofreading in the preparation of this manuscript.

### Author Contributions

Conceptualization, Yayoi Tetsuou Tsukada. and Mashiro Yasutake; methodology, Yayoi Tetsuou Tsukada; scoping review, Yayoi Tetsuou Tsukada and Ritsuko Okamura; validation, Ritsuko Okamura; writing―original draft preparation, Yayoi Tetsuou Tsukada. All authors have read and agreed to the published version of the manuscript.

### Conflicts of Interest

None

### Institutional Review Board Statement

This study was conducted in accordance with the Declaration of Helsinki and approved by the Ethics Committee of Nippon Medical School (no. 626-3-21). All participants and participating companies provided informed consent.

### Data Availability Statement

The data presented in this study are available on request from the corresponding author. The data are not publicly available because all responses were provided in Japanese only.

## Supplement

Supplementary Material Summary of findings tables of the scoping review are also available.
