# Supplementary material for: Comprehensive Health Challenges and Management Strategies for International Business Travelers: A Scoping Review
Source: JMA J. 2025 Sep 5;8(4):1021–30. doi: 10.31662/jmaj.2025-0085 (PMC12598141; doi:10.31662/jmaj.2025-0085)
Supplement: Supplementary Material — Summary of findings tables of the scoping review are also available. [file 2433-3298-8-4-1021-s001.pdf]

Summary of findings table

No	Classification	Title	Year	Journal	Authors, country, year	DOI	Study design	Study objective	Population studied	Outcome	Main finding
1	Adiposity	Association Between Business Travel, Health-Related Behaviors, and Adiposity.	2021	Journal of Occupational and Environmental Medicine	Bergquist SH et.al<br>US, 2021	10.1097/JOM.0000000000002278	retrospective observational study (cross-sectional analysis)	To evaluate the associations between frequency of business travel and health behaviors and adiposity.	795 corporate physical exams.	BMI, body fat percentage, and visceral adipose tissue	In male population, international travel frequency has a greater influence on adiposity than summed (mostly domestic) travel.
2	Corporate Health Management	A Survey on the Current Status of Health Care for Business Travelers Traveling Abroad for Less than 6 months.	2021	Journal of the Japanese Society of Travel and Health (In Japanese)	Kawashima M et.al<br>Japan, 2021	NA	Questionnaires survey	To clarify the actual situation by conducting a questionnaire survey on the current status of health management for overseas business travelers who are dispatched for less than six months.	122 corporate health managers.<br>112 people responded	Collecting medical information.<br>Providing a designated contact point for health consultation.<br>Conducting pre-trip health evaluations.<br>Recommending vaccinations.<br>Setting maximum trip frequencies.<br>Managing overtime during travel.<br>Offering travel insurance.<br>Handling emergency room transport.<br>Addressing recurring mental health issues.<br>Providing worker's compensation.	Around 67.0% of respondents collect medical information during overseas business trips, while 37.5% consider health when deciding on trips. About 31.3% make trips twice a month or more. Nearly half have travel insurance, and 30.3% report mental health issues, with 27.4% experiencing work-related accidents. Approximately 70% collect medical info on trip destinations, and 40% consider health for business trips. However, coordination between health and labor management is lacking, highlighting a need for better integration.
3	Corporate Health Management	Differences in Understanding of Health Care Support Information of Overseas Business Travelers Between Medical and Non-medical Professionals.	2021	Journal of the Japanese Society of Travel and Health (In Japanese)	Kawashima M et.al<br>Japan, 2021	NA	Questionnaires survey	To examine the differences in understanding of the current situation regarding health management of overseas business travelers between medical and non-medical staff, and to clarify the issues for overseas business travelers whose dispatch period is less than 6 months.	204 overseas business travelers.<br>(96 males 103 females, 5 unknown)<br>117 medical professionals, 67 non-medical professionals 0 unidentified.	Collecting medical information.<br>Providing a designated contact point for health consultation.<br>Conducting pre-trip health evaluations.<br>Recommending vaccinations.<br>Setting maximum trip frequencies.<br>Managing overtime during travel.<br>Offering travel insurance.<br>Handling emergency room transport.<br>Addressing recurring mental health issues.<br>Providing worker's compensation.	Significant disparities were observed between medical and non-medical personnel in three key areas: frequency of overseas business trips, handling overtime work, and understanding of travel insurance subscriptions. These discrepancies suggest inadequate coordination of information among professions, posing concerns for duty of care and necessitating effective collaboration to support overseas business travelers adequately.
4	Corporate Health Management	Current Status and Issues of Health Management Measures for People Deployed Abroad.	2015	Journal of the Japanese Society of Travel and Health (In Japanese)	Kurita N et.al<br>Japan, 2015	NA	Questionnaires survey	To establish a health management system for employees dispatched overseas, a questionnaire survey was conducted on "awareness of health issues of employees dispatched overseas" and "current status of health management measures" among corporate health management personnel.	123 respondents to the survey, 81 who responded that their companies had employees on overseas assignment were included in the analysis.	Collecting medical information.<br>Providing a designated contact point for health consultation.<br>Conducting pre-trip health evaluations.<br>Recommending vaccinations.<br>Setting maximum trip frequencies.<br>Managing overtime during travel.<br>Offering travel insurance.<br>Handling emergency room transport.<br>Addressing recurring mental health issues.<br>Providing worker's compensation.	The respondents showed a high level of interest in "mental disorders" and "infectious diseases" as health problems of expatriates, and many of them indicated "medical institutions" and "infectious diseases" as necessary information. Many companies have implemented health management measures for expatriates, but few companies have implemented such measures for business travelers. Based on this situation, it is necessary to establish health management measures for employees dispatched overseas.
5	Infectious disease (COVID-19)	Occupational Safety and Health Challenges for Maritime Key Workers in the Global COVID-19 Pandemic.	2022	International Labour Review	Shan D<br>Switzerland, 2022	10.1111/ilr.12220	Interview (qualitative research methods)	To explore the consequences for seafarers of the COVID-19 pandemic and the related public health measures.	Seafarers 16, Ship managers 5,Union representatives 4, Key informants 4	Impacts of COVID-19 on employment;<br>Health and safety challenges faced by seafarers during extended service at sea<br>Impacts of public health measures on crew changes and shore leave<br>Resources and support available to seafarers during the pandemic.	The pandemic has increased seafarer fatigue and increased the risk of fatigue-related accidents.the weakness of the current international labour governance system, particularly in terms of its limitations in protecting seafarers as essential workers in international transportation.
6	Infectious disease (pre-travel consultation)	Characteristics and Potential Quality Indicators for Evaluating Pre-travel Consultations in Japan Hospitals: the Japan Pretravel Consultation Registry (J-PRECOR).	2022	Tropical Diseases, Travel Medicine and Vaccines	Yamamoto K et.al<br>Japan, 2022	0.1186/s40794-021-00160-4	Retrospective cohort study	To assess the pre-travel consultations(PTC) implementation rate and examined the indicators of PTCs that can be used as criteria for evaluating quality.	9700 eligiblewho presented for their PTCs at 17 facilities and were registered	Medical information was extracted retrospectively via a web-based system. Correlations between vaccination risk categories and advice/intervention proportions by the facility	Travel was predominantly for business (40.5%), with the US (11.5%) and Asia (41.3%) being common destinations. Vaccine numbers increased after pre-travel consultations, except for tetanus toxoid. Only 60.8% recommended for malaria prophylaxis received anti-malarial agents. Factors like gross national income and disease incidence were associated with vaccine administration and prophylactic prescriptions.
7	Infectious disease (pre-travel consultation)	Changing Trends and Pretravel Preparation of Business Travelers from Greece during the Financial Crisis.	2019	Public Health	Pavli A et.al<br>Greece, 2019	10.1016/j.puhe.2018.12.015	Questionnaires survey	To assess the changing trends of business travelers and their pretravel preparation.	A total of 12,379 travelers completed the questionnaire, 58% of whom were business travelers.	Vaccine and malaria prophylaxis travel characteristics	Business travelers, mostly men aged around 34, sought pretravel advice about 18.5 days before departure. Top destinations were the Middle East (47.8%) and Sub-Saharan Africa (28.3%). Most stayed in urban areas (77.6%) for over a month (68.6%). Yellow fever vaccine was common (75%), with notable uptake of other vaccines like typhoid fever (26.9%) and hepatitis A (15.5%). Malaria prophylaxis was administered to 26.8%, especially for Sub-Saharan Africa (46.5%) and the Indian subcontinent (53.5%).
8	Infectious disease (pre-travel consultation)	Pre-Travel Medical Preparation of Business and Occupational Travelers<br>An Analysis of the Global TravEpiNet Consortium, 2009 to 2012	2016	Journal of Occupational and Environmental Medicine	Khan N et.al<br>US, 2016	10.1097/JOM.0000000000000602	A questionnaire-based survey	To understand more about pre-travel preparations and itineraries of business and occupational travelers.	23,534 travelers who visited travel clincs, 61% were non-occupational and 39% occupational.	pre-travel health consultation<br>vaccination	Business travelers, mostly men, had short lead times and trip durations. They commonly declined vaccines for influenza, meningococcal, and hepatitis B. Employers typically recommended pre-travel health consultations for them, while non-occupational travelers sought consultations for personal health reasons.
9	Infectious disease (Vaccine)	Vaccination Status and Awareness Questionnaire for Relatively Short-term Medical Cooperation Travel.	2020	Journal of the Japanese Society of Travel and Health (In Japanese)	Kitoh T et.al<br>Japan, (2020)	NA	Questionnaires survey	To clarify the vaccination status and attitudes toward travel vaccines among health care workers who provide medical cooperation overseas.	29 respondents: 19 had participated in the program for the first time, 4 for the second or third time, and 6 for more	Number of trips, vaccination status , vaccination information, reasons for vaccination. reasons for not receiving the vaccine.	Most participants, primarily in their 20s or 30s, were first-time travelers. Hepatitis A vaccine was received by 34%, hepatitis B by over 75%, tetanus and Japanese encephalitis by over 67%. Rabies vaccine was given to about 25%, and typhoid to 7%. Dental hygienists and speech-language pathologists had lower vaccination rates. Reasons for not vaccinating included inconvenience, lack of awareness, low perceived risk, cost concerns, and prior vaccination history.
10	Infectious disease (Vaccine)	Vaccination and Malaria Prophylaxis among Greek International Travelers to Asian Destinations.	2015	Journal of Infection and Public Health	Pavli A et.al<br>UK, 2015	10.1016/j.jiph.2014.07.002	A questionnaire-based survey	To investigate the pre-travel health-seeking practices of travelers to Asian destinations.	1666 adult travelers (visiting friends and relatives (VFRs) and business travellers)	Pre-travel preparationin in terms of vaccinations and malaria prophylaxis.<br>the decision to seek a pre-travel consultation	Most participants traveled for less than a month (51.4%) and only a quarter pursued pre-travel consultations. Vaccinations were received by 14.4% of participants, with hepatitis A, tetanus/diphtheria, and typhoid being the most common. Malaria prophylaxis was given to 12.2%. Logistic regression analysis found that male gender, unemployment, low education level, travel to visit friends or relatives, and short travel durations were significant factors for not seeking pre-travel consultations.
11	Infectious disease (Vaccine)	Characteristics and Preparation of the Last-minute Traveler: Analysis of Vaccine Usage in the Global TravEpiNet Consortium.	2019	Journal of Travel Medicine	Yates JA et.al<br>US, 2019	10.1093/jtm/taz031	Retrospective observational study	To obtain a better understanding of Last-minute travellers(LMT: travellers with a departure date of 7 days or fewer from the medical encounter)	12494 LMTs who presented to a network of US clinical practices for pre-travel health advice	Pretravel vaccination	LMTs, who comprised 16% of all travelers, were more likely than short-term travelers to travel for business or visit friends and relatives, stay longer than a month, and visit only urban areas. Additionally, 18% of LMTs deferred at least one necessary travel vaccine, often due to insufficient time before departure, with multi-dose vaccines like those for Japanese encephalitis and rabies being most frequently postponed.
12	Infectious disease （KAP)	Knowledge, Attitude and Compliance towards Travel Vaccines among Nigerian Travellers at an International Airport.	2019	African Journal of Primary Health Care & Family Medicine	Akodu BA et.al<br>South Africa, 2019	10.4102/phcfm.v11i1.2063	interviewer-administered questionnaire	To determine the level of knowledge, attitude and compliance to travel vaccines.	198 Nigerian travellers at Murtala Mohammed International Airport, Ikeja, Nigeria.	knowledge of travel vaccines,positive attitude, vaccination for yellow fever before travel	Significant associations exist between tribe, religion, education and knowledge of travel vaccines.
13	Infectious disease （KAP)	Travel Health Attitudes among Turkish Business Travellers to African Countries.	2016	Travel Medicine and Infectious Disease	Selcuk EB et.al<br>Netherlands, 2016	10.1016/j.tmaid.2016.09.005	A questionnaire-based survey	To evaluate the attitudes and health risk awareness of Turkish travellers travelling to African countries	124 Turkish travellers bound for Africa from Istanbul International Ataturk Airport (Worker 60,Businessman 58,Government employee 6)	Travel health behaviours, Knowledge level about infections in the destination, vaccination	Turkish travelers to Africa display significant gaps in vaccination and health risk awareness. Only 2.9% had destination information, with 11.3% seeking health-related travel information. About 53.2% had at least one vaccination, primarily for typhoid. Many lacked awareness of yellow fever vaccination (69.3%) and malaria prophylaxis (80.6%). Higher education levels correlated with receiving recommended vaccinations for the destination.
14	Infectious disease （KAP)	Pre-travel Preparation Practices among Business Travellers to Tropical and Subtropical Destinations: Results from the Athens International Airport Survey.	2014	Travel Medicine and Infectious Disease	Pavli A et.al<br>Netherlands, 2014	10.1016/j.tmaid.2013.12.004	A questionnaire-based survey	To assess travel health preparation practices of business travellers departing to Africa, the Middle East and Asia.	A total of 684 business travellers participated in the study; the majority were men (86.1%), of Greek nationality (95.3%), with tertiary education (90.8%) and employed (98%).	Vaccination<br>Malaria chemoprophylaxis<br>pursuing health information among business travellers.	The most frequent travel destinations were the Middle East (46.8%) and sub-Saharan Africa (16%). Only 58.8% of travelers sought pre-travel health consultation, and of those, 24.7% were vaccinated while 25.7% received malaria chemoprophylaxis. Notably, vaccination rates for Hepatitis A and typhoid were lower than expected at 70% and 35%, respectively, and nearly half of the travelers to malaria-endemic areas did not receive any chemoprophylaxis. Factors such as elementary education, travel to the Middle East or North Africa, short trips, and staying in hotels or houses were associated with skipping health consultations.
15	Jet lag	Eastward Jet Lag is Associated with Impaired Performance and Game Outcome in the National Basketball Association.	2022	Frontiers in Physiology	Leota J et.al<br>Switzerland, 2022	10.3389/fphys.2022.892681	Retrospective observational study	To examine whether the direction of travel-related jet lag is associated with performance in the National Basketball Association (NBA), and if so, to explore potential mechanisms.	Proffesional athletes（NBA players）	Winning percentage, point differential, rebound differential, and effective field goal percentage	Sleep and circadian rhythm disruptions following eastward travel may have significant negative effects on performance in the NBA, especially when recovery time is limited
16	Jet lag	Jet-Lag Countermeasures Used by International Business Travelers.	2021	Aerospace Medicine and Human Performance	Rigney G et.al<br>US, 2021	10.3357/AMHP.5874.2021	A questionnaire-based study	To investigate international business travelers use of jet-lag countermeasures.	107 International business travelers from Australia	Duration of stay and traveling experience on jet-lag countermeasure use	Education programs delivered through businesses would be beneficial for providing information on jet lag
17	Jet lag	Lifestyle Factors and Jet Lag Prevention: a Preliminary Cross-sectional Analysis of Travel Wellness among Japanese and US Business Class Travelers	2021	Sleep and Biological Rhythms	Hayashi H et.al<br>Japan, 2021	10.1007/s41105-020-00297-3	Questionnaires survey	To examine whether behavioral and mental health-related determinants were associated with severity of jet lag and symptoms of jet lag.	1759 Japanese and 483 U.S. participants.	Jet lag perceptions and symptoms of jet lag	A positive association between severity of jet lag perception and sleep disorders, but a negative association between severe jet lag perception and both smoking and physical activity. Regarding jet lag symptoms, it revealed a positive association between higher jet lag symptoms and perceived stress, sleep disorder, and vegetable consumption. The results varied for other lifestyle factors, including smoking, physical activity, vegetable intake, and alcohol consumption.
18	Travel-related illnesses and injury	Health Risk Perceptions and Behaviors among Japanese Overseas Workers Serving in Low- and Middle-income Countries: A Qualitative Study	2022	Journal of International Health	Sasayama K et.al<br>Japan, 2022	10.11197/jaih.37.69	Interview	To investigate health risk perception, health behavior, and disease prevention among Japanese overseas workers who had lived in low and middle-income countries.	8 Japanese experienced in sponsored overseas volunteer work (3 men; 5 women)	Pre-overseas travel concerns,<br>increased awareness of travel related risks desire to avoid injury or illness.	The health risk perceptions of the overseas worker were informed by their past experience and from pre-departure advice, which in turn influenced their pre-departure preparation. Pre-departure and in-country advice could be very important to support the efforts of overseas workers to avoid injury or illness
19	Travel-related illnesses and injury	Business Travel-associated Illness: a GeoSentinel Analysis.	2018	Journal of Travel Medicine	Chen LH et.al<br>UK, 2018	10.1093/jtm/tax097	Retrospective observational study	To describe travel-related health problems in business travelers	12,203 business travelers with 14,045 eligible diagnoses<br>(10 567 non-expatriate business travelersb with 12 080 total diagnoses)	Analysis covered demographics, travel itineraries, diagnoses, and seriousness of conditions for business travelers.Distinguished between expatriate and non-expatriate business travelers' diagnoses.	Among non-expatriate business travelers, the most common diagnoses were acute unspecified diarrhea, viral syndrome, acute bacterial diarrhea, chronic diarrhea, and P. falciparum malaria (each <10%). P. falciparum malaria was frequent in sub-Saharan Africa (13%), and P. vivax malaria in Oceania (12%). The Caribbean saw the most cases of dengue infection (9%), while dog bites were common in Eastern Europe (9%). Upper respiratory tract infection was most common in Australia, New Zealand, North America, and Western Europe (8%). Acute unspecified diarrhea was the leading diagnosis in other regions (10%-19%).
20	Travel-related illnesses and injury	Medical Evacuations in the Oil and Gas Industry: a Retrospective Review with Implications for Future Evacuation and Preventative Strategies.	2017	Journal of Travel Medicine	Toner S et.al<br>UK, 2017	10.1093/jtm/taw095	Retrospective observational study	To develop a remote healthcare strategy whereby enhanced remote healthcare made available to the patient through use of telemedicine and telemetry	Review of Medevacs data of 31 860 Shell International employees [i.e. expatriate employees (EEs) and frequent business travellers (FBTs).<br>Employee records and Human Resource data were used as a denominator for the population.	Basic demographic information, the incident country and corresponding country risk categorization, and ICD-10 description and codes for the medical diagnoses for Medevac. Further detail was added to this with regard to any medical histories of employees available through employee records held by Human Resources (HR)	Female business travellers were at higher risk of Medevac than male business travellers.There was evidence of an age bias. Medevac rates were highest for Africa and countries classified as 'high-risk'.The diagnoses of a digestive and traumatic nature were most frequently associated with Medevac. Illness was a more prevalent cause of Medevac than traumatic injury.
21	Travel-related illnesses and injury	Assessing the Risk of Work-related International Travel.	2014	Journal of Occupational and Environmental Medicine	Druckman M et.al<br>US, 2014	10.1097/JOM.0000000000000314	Retrospective observational study	To identify factors affecting the likelihood of requiring medical services during international business trips.	Claim data from more than 800,000 international trips and medical assistance cases provided to 48 multinational corporations in 2009.	Hospitalizations, medical evacuations,risk per trip	Travel to "low" medical risk countries in aggregate accounted for more hospitalizations and medical evacuations than travel to "high" medical risk countries. Nevertheless, the risk per trip was much higher for travel to higher medical risk countries.
22	Well-being	Ill-being or Well-being? Energising International Business Travellers	2020	Journal of Organizational Effectiveness: People and Performance	Rattrie L et.al<br>UK, 2020	10.1108/JOEPP-02-2019-0011	Semi-structured in-depth interviews	To explore well-being experiences of international business travellers (IBTs) and contribute to our understanding of personal and job characteristics as antecedents of ill- or well-being.	32 IBTs assigned to various destinations ranging from single-country travel to global operation.	Work-related outcomes such as stress and burnout or health and well-being	The concept of trip-load and intensity of travel are seen as factors along a spectrum affecting work-related outcomes like stress or well-being. These factors are moderated by cognitive and behavioral traits. The findings propose a framework for enhancing the well-being of international business travelers (IBTs) by adjusting job and travel characteristics, alongside providing guidance for training and development to empower IBTs in self-management.
23	Well-being	Stress of Working Abroad: a systematic Review.	2018	International Archives of Occupational and Environmental Health	Doki S et.al<br>Germany, 2018	10.1007/s00420-018-1333-4	Systematic review.	To identify the stress factors affecting foreign-born worker	Workers living abroad, such as immigrants, expatriates and business travellers	Workers' stress and stress-related illness (e.g. depressive mood, fatigue, exhaustion, burnout, irritation, depression, anxiety and sleep disorder).	For the systematic review, 45 out of 14,994 articles were analysed. Six components, communication, cultural differences in the workplace, daily life, relationships with family and colleagues, financial problems and social inequality, were extracted.
24	Well-being	Business Travel and Behavioral and Mental Health.	2018	Journal of Occupational and Environmental Medicine	Rundle AG et.al<br>US, 2018	10.1097/JOM.0000000000001262.	Retrospective observational study	To assess associations between business travel and behavioral and mental health	De-identified electronic medical record data	Smokimg, sleeping, be sedentary, alchole dependence, anviety, depression	Frequent business travelers were more likely to smoke, experience trouble sleeping, be sedentary, and have higher rates of alcohol dependence, anxiety, and depression.
25	Well-being	A Preliminary Questionnaire Study of Long-haul Flight Crew Fatigue.	2017	Labor Science	Sasaki T et.al<br>Japan, 2017	10.11355/isljsl.93.116	Questionnaires survey	To clarify what fatigue means to pilots on short- and long-haul flights (SHF and LHF, respectively).	Four airlines employed a total of 3,436 pilots.	The perceived causes of fatigue<br>its signs and symptoms in the reporting pilot and observed in others<br>the strategies used to minimize its impact.	Both long-haul (LHF) and short-haul (SHF) pilots experience fatigue, with different triggers. LHF fatigue is mainly attributed to night flights and jet lag, while SHF fatigue stems from prolonged duty periods and early wake-ups. Both groups report reduced alertness, attention, and concentration. Fatigue affects flying tasks, increasing difficulty. Rest and sleep management are primary coping strategies for both LHF and SHF pilots. Duty time is a significant predictor of fatigue, intertwined with other contributing factors such as work schedules and sleep deprivation.
26	Well-being/Leadership	International Business Travelers' Job Exhaustion: Effects of Travel Days Spent in Short-haul and Long-haul Destinations and the Moderating Role of Leader-member Exchange	2021	Journal of Global Mobility-The Home of Expatriate Management Research	Mäkelä L et.al<br>Finland, 2021	10.1108/JGM-10-2020-0066	Questionnaires survey	To acess study the association of the duration of international business travel To short-haul and long-haul destinations (travel days/past 12 months) and the Total number of travel days on exhaustion related To international business travel and leadership.	570 emplyees of Finnish software and service company operating in several countries and employing over 13,000 people.	International business traveling related job exhosting<br>Leader-member exchange	The results suggest that a higher number of international business travel days is related to a higher level of job exhaustion, especially the exhaustion related to international business travel. Moreover, a high-quality leadership was found to be linked to lower levels of both types of exhaustion. Interestingly, for those IBTs' with a low-quality leadership, even a high number of long-haul international business travel days was not connected with IBT-specific job exhaustion.
27	Work style	When the Impossible Becomes Possible: COVID-19's Impact on Work and Travel Patterns in Swedish Public Agencies	2021	European Transport Research Review	Hiselius LW et.al<br>Sweden, 2021	10.1186/s12544-021-00471-9	Questionnaires survey	To analyse the effects of government and public agencies' recommendations on meeting and travel behaviour on employees at five public agencies in Sweden.	719 answers out of 1020 employees at five public agencies in Sweden<br>a total of were received	Commuting and business travel<br>attitude toward and choice of travel modes<br>the use of telework and virtual collaboration<br>questions relating to these aspects before and during the time of the survey	The public authorities surveyed were well prepared and had a 'backup collaboration solution', at least technically, to make a rapid behavioural shift when travel was not an option. Though the Swedish government's and Public Health Authority's strong recommendations have led to the most dramatic reductions in work-related travel in modern times, the operations in Swedish agencies continue to function, along with the employees' communications and collaborations.
28	Work style	Videoconferencing and Business Air Travel: Do New Technologies Produce New Interaction Patterns?	2013	Transportation Research Part C: Emerging Technologies	Denstadli JM<br>Norwar, 2013	10.1016/j.trc.2012.12.009	Questinnar survey	To summarize the emergence of videoconferencing as a business communication tool and emphasize the most recent developments	Business air passengers	Videoconferencing usage	The relationship between videoconferencing and business air travel appears generally positive, with frequent air travelers often participating in numerous video meetings. Nevertheless, significant variations exist in travel and videoconferencing usage, with a notable portion of respondents considering travel substitution. Regression analysis suggests that those with access to in-house videoconferencing rooms are more inclined towards substitution compared to those solely reliant on "new" technologies, even when accounting for individual and company factors.
29	Work style/ Well-being	The Dark Side of Business Travel: A Media Comments Analysis.	2018	Transportation Research Part D: Transport and Environment	Cohen S et.al<br>UK, 2018	10.1016/j.trd.2017.01.004	Qualitative method of analysis	To acess the personal and social consequences of frequent travel	150,000 social media shares and 433 media comments from readers,	Well-being	The analysis finds two key identities are performed through public responses to the explicit health and social warnings concerned with frequent business travel: the 'flourishing hypermobile' and the 'floundering hypermobile'. The former either deny the health implications of frequent business travel, or present strategies to actively overcome them, while the latter seek solace in the public dissemination of the health warnings: they highlight their passivity in the construction of their identity as hypermobile and its associated health implications.
30	Work style/ Well-being	Associations Between Work Family Conflict, Emotional Exhaustion, Musculoskeletal Pain, and Gastrointestinal Problems in a Sample of Business Travelers.	2015	Scandinavian Journal of Psychology	Jensen MT et.al<br>UK, 2015	10.1111/sjop.12177	Questionnaire survey	To examine the associations among work-family conflict (WFC), emotional exhaustion, musculoskeletal (MS) pain, and gastrointestinal problems on a sample of business travelers . Additionally to examine differences in the mentioned relationships among three traveler groups: commuters, national travelers, and international travelers	3078 business travelers.Travel pattern consisted of three traveler groups: Commuters (153), national travelers (1,434), and international travelers (506).	Emotional exhaustion<br>Musculoskeletal pain<br>Gastrointestinal problems	Positive relation between Work-Family conflict and emotional exhaustion.Work-Family conflict did not relate to musculoskeletal pain or gastrointestinal problems. emotional exhaustion was related positively to both musculoskeletal pain and gastrointestinal problemsthe association between emotional exhaustion and musculoskeletal pain proved to be significantly stronger for the commuter group compared to the national and international travel groups
31	Work style/ Well-being	Family to Work Conflict and the Usefulness of Workplace Support.	2013	Occupational Medicine-Oxford	Hancock F<br>UK, 2013	10.1093/occmed/kqt053	Online anonymous survey	To investigate employee perceptions about the effect of family to work conflict (FWC) on work.	286 employees at a UK private sector research and development plant	Family and other domestic stressors.<br>Work effects including on business travel, work performance and the awareness and usefulness of work-provided support.	Among the respondents, 86% of men and 65% of women were business travelers, with 27% of men and 11% of women traveling frequently. FWC led to reduced business trips for 13% and affected work performance for 29% of men and 17% of women, especially frequent travelers. Support was valuable across FWC: 58% appreciated supervisor support, 52% colleague support, and 31% occupational health (OH). Those with FWC affecting work performance found colleague, line-manager , and OH support particularly helpful. Only 53% knew of the Employee Assistance Program (EAP), with 19% using it (58% were female). More women sought EAP advice (30%) compared to men.
